# Supplementary material for: From sequence to enzyme mechanism using multi-label machine learning
Source: BMC Bioinformatics. 2014 May 19;15:150. doi: 10.1186/1471-2105-15-150 (PMC4229970; doi:10.1186/1471-2105-15-150)
Supplement: Additional file 2 — Java code of ml2db. Additional file ml2db_code.tar.gz contains the Java source code to run the multi-label machine learning experiments and save the results to database. The code’s Javadoc is included. [file 1471-2105-15-150-S2.zip › additional file 2/ml2db/ml2db/doc/main/ml2db/ProjectParameters.html]

ProjectParameters


JavaScript is disabled on your browser.


- Overview
- Package
- Class
- Use
- Tree
- Deprecated
- Index
- Help

- Prev Class
- Next Class

- Frames
- No Frames

- All Classes

- Summary:
- Nested |
- Field |
- Constr |
- Method

- Detail:
- Field |
- Constr |
- Method


main.ml2db

## Class ProjectParameters

- java.lang.Object
- - main.ml2db.ProjectParameters

- Direct Known Subclasses:
  :   TestProjectParameters

  ---

    

  ```
  public class ProjectParameters
  extends java.lang.Object
  ```

  Contains file paths (database connection files, arff properties files, mulan
  xml files etc.)

  Version:
  :   14 Feb 2014

  Author:
  :   Luna De Ferrari ldf(at)st-andrews.ac.uk

- - ### Field Summary

    Fields

    | Modifier and Type | Field and Description |
    | --- | --- |
    | `static java.lang.String` | `ARFF_FILES_DIR` |
    | `static java.lang.String` | `ARFF_FILES_PATH` |
    | `static java.lang.String` | `ARFF_PROPS_PATH` |
    | `static java.lang.String` | `ARFF_TABLE` The name of the table to save the arff file details to |
    | `static java.lang.String` | `AUTHOR` |
    | `static java.lang.String` | `DATA_LOCAL_DB` |
    | `static java.lang.String` | `DATA_REMOTE_DB` |
    | `static int` | `EXPECTED_NUMBER_OF_UNIQUE_ATTRIBUTES` used to optimise the pool of attributes strings |
    | `static int` | `EXPECTED_NUMBER_OF_UNIQUE_INSTANCES` used to optimise the pool of instances strings |
    | `static java.lang.String` | `FIGURES_PATH` |
    | `static java.lang.String` | `MACHINE_LEARNING_LOCAL_DB_PROPS` |
    | `static java.lang.String` | `MACHINE_LEARNING_PROPS` |
    | `static java.lang.String` | `MACHINE_LEARNING_REMOTE_DB_PROPS` |
    | `static java.lang.String` | `MACIE_HOM_DB_PROPS` |
    | `static java.lang.String` | `MODELS_PATH` |
    | `static java.lang.String` | `PREDICTIONS_PATH` |
    | `static java.lang.String` | `PROJECT_NAME` |
    | `static java.lang.String` | `RESULTS_DIRECTORY` |
    | `static java.lang.String` | `RESULTS_DIRECTORY_ACTIVE_LEARNING` |
    | `static java.lang.String` | `SOFTWARE_PROJECT_NAME` |
    | `static java.lang.String` | `STATISTICS_PATH` |
    | `static java.lang.String` | `UNIPROT_DB_PROPS` |
    | `static java.lang.String` | `VERSION` |
    | `static java.lang.String` | `WEB_PAGE` |
  - ### Constructor Summary

    Constructors

    Constructor and Description

    `ProjectParameters()`
  - ### Method Summary

    Methods

    | Modifier and Type | Method and Description |
    | --- | --- |
    | `static DbManagers` | `getLocalDbManagers()` |
    | `static DbManagers` | `getRemoteDbManagers()` |

    - ### Methods inherited from class java.lang.Object

      `equals, getClass, hashCode, notify, notifyAll, toString, wait, wait, wait`

- - ### Field Detail


    - #### MODELS\_PATH

      ```
      public static final java.lang.String MODELS_PATH
      ```

      See Also:
      :   Constant Field Values


    - #### FIGURES\_PATH

      ```
      public static final java.lang.String FIGURES_PATH
      ```

      See Also:
      :   Constant Field Values


    - #### PREDICTIONS\_PATH

      ```
      public static final java.lang.String PREDICTIONS_PATH
      ```

      See Also:
      :   Constant Field Values


    - #### STATISTICS\_PATH

      ```
      public static final java.lang.String STATISTICS_PATH
      ```

      See Also:
      :   Constant Field Values


    - #### PROJECT\_NAME

      ```
      public static final java.lang.String PROJECT_NAME
      ```

      See Also:
      :   Constant Field Values


    - #### ARFF\_FILES\_DIR

      ```
      public static final java.lang.String ARFF_FILES_DIR
      ```

      See Also:
      :   Constant Field Values


    - #### ARFF\_FILES\_PATH

      ```
      public static final java.lang.String ARFF_FILES_PATH
      ```

      See Also:
      :   Constant Field Values


    - #### ARFF\_PROPS\_PATH

      ```
      public static final java.lang.String ARFF_PROPS_PATH
      ```

      See Also:
      :   Constant Field Values


    - #### RESULTS\_DIRECTORY

      ```
      public static final java.lang.String RESULTS_DIRECTORY
      ```

      See Also:
      :   Constant Field Values


    - #### RESULTS\_DIRECTORY\_ACTIVE\_LEARNING

      ```
      public static final java.lang.String RESULTS_DIRECTORY_ACTIVE_LEARNING
      ```

      See Also:
      :   Constant Field Values


    - #### MACHINE\_LEARNING\_LOCAL\_DB\_PROPS

      ```
      public static final java.lang.String MACHINE_LEARNING_LOCAL_DB_PROPS
      ```

      See Also:
      :   Constant Field Values


    - #### MACHINE\_LEARNING\_REMOTE\_DB\_PROPS

      ```
      public static final java.lang.String MACHINE_LEARNING_REMOTE_DB_PROPS
      ```

      See Also:
      :   Constant Field Values


    - #### UNIPROT\_DB\_PROPS

      ```
      public static final java.lang.String UNIPROT_DB_PROPS
      ```

      See Also:
      :   Constant Field Values


    - #### MACIE\_HOM\_DB\_PROPS

      ```
      public static final java.lang.String MACIE_HOM_DB_PROPS
      ```

      See Also:
      :   Constant Field Values


    - #### DATA\_REMOTE\_DB

      ```
      public static final java.lang.String DATA_REMOTE_DB
      ```

      See Also:
      :   Constant Field Values


    - #### DATA\_LOCAL\_DB

      ```
      public static final java.lang.String DATA_LOCAL_DB
      ```

      See Also:
      :   Constant Field Values


    - #### MACHINE\_LEARNING\_PROPS

      ```
      public static final java.lang.String MACHINE_LEARNING_PROPS
      ```

      See Also:
      :   Constant Field Values


    - #### ARFF\_TABLE

      ```
      public static final java.lang.String ARFF_TABLE
      ```

      The name of the table to save the arff file details to

      See Also:
      :   Constant Field Values


    - #### AUTHOR

      ```
      public static final java.lang.String AUTHOR
      ```

      See Also:
      :   Constant Field Values


    - #### SOFTWARE\_PROJECT\_NAME

      ```
      public static final java.lang.String SOFTWARE_PROJECT_NAME
      ```

      See Also:
      :   Constant Field Values


    - #### VERSION

      ```
      public static final java.lang.String VERSION
      ```

      See Also:
      :   Constant Field Values


    - #### WEB\_PAGE

      ```
      public static final java.lang.String WEB_PAGE
      ```

      See Also:
      :   Constant Field Values


    - #### EXPECTED\_NUMBER\_OF\_UNIQUE\_ATTRIBUTES

      ```
      public static final int EXPECTED_NUMBER_OF_UNIQUE_ATTRIBUTES
      ```

      used to optimise the pool of attributes strings

      See Also:
      :   Constant Field Values


    - #### EXPECTED\_NUMBER\_OF\_UNIQUE\_INSTANCES

      ```
      public static final int EXPECTED_NUMBER_OF_UNIQUE_INSTANCES
      ```

      used to optimise the pool of instances strings

      See Also:
      :   Constant Field Values
  - ### Constructor Detail


    - #### ProjectParameters

      ```
      public ProjectParameters()
      ```
  - ### Method Detail


    - #### getLocalDbManagers

      ```
      public static DbManagers getLocalDbManagers()
      ```


    - #### getRemoteDbManagers

      ```
      public static DbManagers getRemoteDbManagers()
      ```


- Overview
- Package
- Class
- Use
- Tree
- Deprecated
- Index
- Help

- Prev Class
- Next Class

- Frames
- No Frames

- All Classes

- Summary:
- Nested |
- Field |
- Constr |
- Method

- Detail:
- Field |
- Constr |
- Method
